# Supplementary material for: A Critical View of the Application of the APEX Software (Aqueous Photochemistry of Environmentally-Occurring Xenobiotics) to Predict Photoreaction Kinetics in Surface Freshwaters
Source: Molecules. 2019 Dec 18;25(1):9. doi: 10.3390/molecules25010009 (PMC7017383; doi:10.3390/molecules25010009)
Supplement: Supplementary file 1 [file molecules-25-00009-s001.zip › 3.2.4_gcc-4.4.0.zip!/3.2.4_gcc-4.4.0/3.2.4_gcc-4.4.0/doc/octave/html/interpreter/Processing-Data-in-Structures.html]

Processing Data in Structures - Untitled


Previous: Manipulating Structures,
Up: Data Structures


---

#### 6.1.5 Processing Data in Structures

The simplest way to process data in a structure is within a `for`
loop (see Looping Over Structure Elements). A similar effect can be
achieved with the `structfun` function, where a user defined
function is applied to each field of the structure.

— Function File: **structfun** (func, s)  
— Function File: [a, b] = **structfun** (...)  
— Function File: **structfun** (..., "ErrorHandler", errfunc)  
— Function File: **structfun** (..., "UniformOutput", val)  
> Evaluate the function named name on the fields of the structure
> s. The fields of s are passed to the function func
> individually.
>
> `structfun` accepts an arbitrary function func in the form of
> an inline function, function handle, or the name of a function (in a
> character string). In the case of a character string argument, the
> function must accept a single argument named x, and it must return
> a string value. If the function returns more than one argument, they are
> returned as separate output variables.
>
> If the parameter "UniformOutput" is set to true (the default), then the function
> must return a single element which will be concatenated into the
> return value. If "UniformOutput" is false, the outputs placed in a structure
> with the same fieldnames as the input structure.
>
> ```
>           s.name1 = "John Smith";
>           s.name2 = "Jill Jones";
>           structfun (@(x) regexp (x, '(\w+)$', "matches"){1}, s,
>                      "UniformOutput", false)
> ```
>
> Given the parameter "ErrorHandler", then errfunc defines a function to
> call in case func generates an error. The form of the function is
>
> ```
>           function [...] = errfunc (se, ...)
> ```
>
> where there is an additional input argument to errfunc relative to
> func, given by se. This is a structure with the elements
> "identifier", "message" and "index", giving respectively the error
> identifier, the error message, and the index into the input arguments
> of the element that caused the error.
>
>
>
> **See also:** cellfun, arrayfun.

Alternatively, to process the data in a structure, the structure might
be converted to another type of container before being treated.

— Built-in Function: **struct2cell** (S)  
> Create a new cell array from the objects stored in the struct object.
> If f is the number of fields in the structure, the resulting
> cell array will have a dimension vector corresponding to
> `[`F `size(`S`)]`.
>
>
>
> **See also:** cell2struct, fieldnames.
